# *Lycii radicis* cortex alleviates fibrosis in hiPSC-derived multilineage hepatic organoids via the cAMP-PKA pathway

**DOI:** 10.3389/fphar.2025.1730255

**Published:** 2025-11-25

**Authors:** Junming Xu, Xiaopu Sang, Yongfei He, Jie Ke, Jiasen Xu, Tanbin Liu, Jicai Wang, Hang Zhai, Xiaoni Chen, Xianjie Shi, Fenfang Wu

**Affiliations:** 1 Department of Hepatobiliary and Pancreatic Surgery, the Eighth Affiliated Hospital of Sun Yat-sen University, Shenzhen, Guangdong, China; 2 Biotherapy Center, Shenzhen Third People’s Hospital, Shenzhen, Guangdong, China; 3 Department of Central Laboratory, Shenzhen Hospital, Beijing University of Chinese Medicine, Shenzhen, Guangdong, China; 4 School of traditional Chinese medicine, Hubei University of Chinese Medicine, Wuhan, Hubei, China; 5 Hubei Shizhen Laboratory, Wuhan, Hubei, China

**Keywords:** natural products, IPSCs (induced pluripotent stem cells), organoid, liver fibrosis, lycii radicis cortex, cAMP–PKA–CREB pathway

## Abstract

**Background:**

Liver fibrosis, driven by excessive extracellular matrix (ECM) deposition and activation of hepatic stellate cells (HSCs), still lacks effective therapies, partly due to the absence of human-relevant models. Lycii Radicis Cortex (LRC), a traditional Chinese medicine, exhibits reported anti-inflammatory and antioxidant activities, yet its anti-fibrotic potential has not been validated in human organoid-based systems.

**Methods:**

We established hiPSC-derived multilineage hepatobiliary organoids (mHBOs) containing mesoderm-derived HSCs and implemented a TGF-β–induced fibrosis model within this platform. Using mHBOs alongside a CCl4-injury mouse model, we assessed the anti-fibrotic activity of LRC, and investigated underlying mechanisms.

**Results:**

LRC significantly attenuated fibrosis in mHBOs and in CCl_4_-injured mice, reducing ECM accumulation and HSC activation. In mHBOs, LRC activated the cAMP–PKA–CREB pathway, thereby suppressing HSC activation and reducing parenchymal apoptosis; these effects were reversed by PKA inhibition.

**Conclusion:**

LRC exhibits potent anti-fibrotic activity in a physiologically relevant human organoid model, providing mechanistic insight into HSC regulation and supporting its potential as a candidate therapy for chronic liver disease. Furthermore, this study introduces a translational platform integrating animal models and hiPSC-derived organoids to facilitate anti-fibrotic drug discovery and evaluation.

## Introduction

Liver fibrosis, characterized by excessive extracellular matrix (ECM) deposition resulting from sustained hepatic injury and activation of hepatic stellate cells (HSCs), is a common outcome of chronic liver diseases and a precursor to cirrhosis, liver failure, and hepatocellular carcinoma with substantial global morbidity and mortality (Kisseleva and Brenner, 2021; Akkız et al., 2024). Although cessation of liver injury may allow partial regression of fibrosis, there are currently no approved anti-fibrotic drugs with proven clinical efficacy (Zhao et al., 2022). Many agents that show efficacy in rodent models fail in clinical trials, underscoring the limited translational relevance of conventional liver models. Recent advances in human induced pluripotent stem cell (hiPSC)-derived organoid technology have provided a more predictive platform for identifying and validating novel anti-fibrotic targets and agents (Verstegen et al., 2025; Huang et al., 2025).

Multilineage hepatic organoids generated from hiPSCs, containing parenchymal hepatocyte-like cells alongside non-parenchymal lineages such as HSC, have emerged as physiologically relevant models capable of mimicking key aspects of liver development, homeostasis, and injury responses (Wu et al., 2019; Shi et al., 2022; Guan et al., 2021; Kim et al., 2023). However, most reported multilineage organoids systems typically rely on separately differentiating each cell lineage to maturity before combining them, a strategy that often results in marked heterogeneity in cellular maturation and functionality. Additionally, such models suffer from considerable batch-to-batch variability and a limited ability to consistently induce and maintain fibrotic responses, which constrains their utility in mechanistic studies and anti-fibrotic drug screening. Based on our previous work (Wu et al., 2019; Shi et al., 2022), we developed multilineage hepatobiliary organoids (mHBOs) construction approach in which multiple cell lineages undergo synchronous maturation and self-organization during co-differentiation, thereby enhancing intercellular communication, establishing physiologically relevant architecture, and improving reproducibility.

*Lycii Radicis* Cortex (LRC, named Digupi in Chinese), derived from the root bark of *Lycium barbarum* L. or related species in the Solanaceae family, has long been used in traditional Chinese medicine for treating chronic inflammatory and liver disease (Yang et al., 2017; Chen et al., 2021; Anwar et al., 2020; Xie et al., 2014). Nevertheless, most current evidence is derived from animal models or conventional cell cultures, which fail to recapitulate the complex human hepatic fibrotic microenvironment and multicellular interactions. The lack of validation in organoid-based models limits precise mechanistic elucidation and hinders clinical translation. Therefore, integrating LRC pharmacological investigation with mHBOs technology could enable systematic evaluation of its anti-fibrotic effects under physiologically relevant conditions that closely mimic human disease progression.

In this study, we sought to bridge these gaps by integrating LRC pharmacological evaluation with a physiologically relevant human mHBO fibrosis model. We combined *in vivo* assessment of a carbon tetrachloride (CCl_4_)-induced mouse model with *in vitro* mHBOs modeling of transforming growth factor (TGF)-β driven fibrosis to systematically investigate the anti-fibrotic potential of LRC. Furthermore, using network pharmacology analyses, we identified putative bioactive compounds, molecular targets, and signaling pathways mediating its effects. Mechanistic studies focused on the cyclic adenosine monophosphate (cAMP)-dependent protein kinase A (PKA) axis revealed that LRC attenuates fibrosis by suppressing parenchymal apoptosis in mHBOs. This work establishes a translational pipeline from animal models to human organoids, providing both mechanistic insight and methodological advancement for anti-fibrotic drug discovery.

## Materials and methods

### Animal experiments

Male Kunming (KM) mice aged 6–8 weeks (Shenzhen TOP Biotechnology Co., Ltd.; license SYXK [Guangdong] 2020–0230) were acclimatized for 1 week and maintained under specific pathogen free conditions on a 12 h light/12 h dark cycle with *ad libitum* access to food and water before experimentation. Fifteen mice were randomly assigned to three groups (Control, Model, LRC; n = 5 per group). Hepatic fibrosis was induced in the Model and LRC groups by intraperitoneal injection of a carbon tetrachloride (CCl_4_, MCE, HY-Y0298)–olive oil (MCE, HY-108749) mixture (CCl_4_:olive oil = 1:1) at 1 mL/kg twice weekly for 6 weeks, while the Control group received intraperitoneal olive oil at 1 mL/kg on the same schedule. From week 4 of CCl_4_ administration, the LRC group received oral LRC (Sinopep-Allsino, 630252095148SY; 50 mg/kg) every other day until week 6, this dose was selected with reference to prior mouse studies in which it is commonly used and non-toxic (Chen et al., 2016; Mughal et al., 2022; Son et al., 2024; Shimato et al., 2020; Lee et al., 2021). The Control and Model groups received vehicle (olive oil) on the same schedule. At the end of week 6, mice were euthanized using sodium pentobarbital (3% solution; Servicebio Technology), and serum and liver tissues were collected for subsequent analyses. Body weight and behavior were monitored weekly throughout the study, and no adverse events or deaths were observed.

### Liver function tests

Blood was collected from the ocular orbit and centrifuged at 13,000 rpm for 10 min to obtain serum. Serum alanine aminotransferase (ALT) and aspartate aminotransferase (AST) were measured using a Mindray BS-420 automatic biochemical analyzer (Shenzhen Mindray Bio-Medical Electronics Co., Ltd.).

### Histological analysis

Liver tissues were fixed in 10% formalin, embedded in paraffin blocks, and sectioned at 5-μm thickness. Hematoxylin and eosin (H&E) staining was performed to assess morphological changes. Hepatic collagen deposition associated with fibrosis was evaluated by Sirius Red and Masson’s trichrome staining.

## Cell culture and mHBOs differentiation

Human iPSC line UC (Wu et al., 2019; Shi et al., 2022) was obtained from the Guangzhou Institutes of Biomedicine and Health, Chinese Academy of Sciences. Passages of the cell lines ranged from 40 to 50. Human iPSC line Q-iPS-10 was purchased from the Institute of Zoology, Chinese Academy of Sciences. Passages of the cell lines ranged from 18 to 23. Cells were cultured on Matrigel-coated plates (Corning, 354,277) in mTeSRplus medium (STEMCELL Technologies, 100–0276) at 37 °C in a humidified incubator (ESCO, CLM-240B-8-CN) with 5% CO_2_. The medium was refreshed daily, and cells were passaged when they reached approximately 80% confluency. The transmission ratio is in the region of 1:6 to 1:8.

Upon reaching approximately 80% confluence, the medium was aspirated and cells were washed once with PBS. Accutase was added, and the cells were incubated for approximately 5 minutes. The digestion was terminated with DMEM/F12. Following centrifugation, the cells were resuspended in CryoStor CS10 cryopreservation medium (STEMCELL Technologies, 100–1061), transferred to freezing tubes tube and a record made. The tubes were placed in a freezing container at −80 °C overnight and subsequently transferred to liquid nitrogen for long-term storage.

We generated mHBOs following previously described protocols (Wu et al., 2019), in which a small fraction of mesodermal cells is intentionally retained during definitive endoderm specification. Under directed induction, endodermal cells differentiate into hepatic parenchymal cells, whereas the mesodermal fraction gives rise to non-parenchymal lineages, including hepatic stellate cells, as described previously.

## Fibrosis induction and treatments in mHBOs

On day 25 of culture, mHBOs were allocated to four groups: Control, Model, LRC, and H89+LRC. Fibrogenesis was induced in the Model and LRC groups by a 96-h continuous exposure to transforming growth factor-β (TGF-β, 20 ng/mL; NovoProtein, CA72); the Control group received an equal volume of phosphate-buffered saline (PBS). From 48 h onward, the LRC group received LRC (50 μg/mL) until endpoint; the Control and Model groups received vehicle (DMSO) on the same schedule. The 50 μg/mL concentration was selected with reference to prior *in vitro* studies across multiple cell types, where it is commonly used and non-cytotoxic (Anwar et al., 2020; Mughal et al., 2022; Son et al., 2024; Lee et al., 2021; Park et al., 2019). In the absence of mHBO-specific reports, this choice follows established cell-based practice. To interrogate involvement of the cAMP–PKA pathway, a fourth experimental group was included that received identical LRC treatment but was pretreated with the PKA inhibitor H89 for 30 min prior to LRC exposure. At the end of treatment, organoids and conditioned media were collected for downstream analyses, including qPCR, immunofluorescence, and ELISA.

## Analysis of qRT-PCR

Total RNA was isolated from cultured cells using the High-Purity RNA Rapid Extraction Kit (MIKX, MKG-865). cDNA was synthesized from total RNA with the PrimeScript RT Reagent Kit with gDNA Eraser (Takara, RR037A) according to the manufacturer’s protocol. qPCR was performed using TB Green Premix Ex Taq II (Takara, RR820A) on a q900 thermal cycler. Relative transcript abundance was calculated using the 2^−ΔΔCT^ method with glyceraldehyde-3-phosphate dehydrogenase (*GAPDH*) as the internal reference. Primer sequences are provided in Supplementary Table S2.

## Immunofluorescence

Cultures were first exposed to 4% paraformaldehyde (PFA; Solarbio, P1110) at room temperature for 30 min, after which membranes were permeabilized for 1 h in DPBS containing 0.3% Triton X-100 (Sigma-Aldrich, T9284). Samples were rinsed three times with DPBS (5 min each) and then placed in 5% donkey serum (Solarbio, SL050) in DPBS for at least 1 h. Primary antibodies diluted in 3% donkey serum were applied for 1 h at room temperature or overnight at 4 °C, followed by secondary antibodies prepared in 1% donkey serum for 30 min at room temperature. Where nuclear labeling was required, DAPI was introduced for 3 min. After the primary, secondary, and DAPI steps, cultures were washed four times with DPBS at room temperature (5 min per wash). At least three samples per group were stained, with three non-overlapping fields of view captured per sample. Images were acquired using a confocal laser scanning microscope (LSM800; Carl Zeiss). The list of antibodies used in this study were provided in Supplementary Table S3.

## Flow cytometry

Cultures were dissociated into single cells with trypsin at 37 °C and pelleted by centrifugation at 350 *g* for 3 min. After three washes with DPBS, 250 µL of Fixation/Permeabilization solution (BD, 554,714) was added, and cells were incubated for 20 min at 4 °C. Cells were then washed twice with BD Perm/Wash™ buffer (BD, 554,714) and resuspended thoroughly in 100 µL of BD Perm/Wash™ containing fluorochrome-conjugated antibodies at empirically determined working dilutions or appropriate isotype/negative controls. Incubations were performed for 30 min at 4 °C in the dark. Following two additional washes with Perm/Wash™ (1 mL per tube each), cells were resuspended in DPBS and subjected to flow-cytometric acquisition. Data were collected on a BD FACS LSRFortessa flow cytometer and analyzed using FlowJo (v10.8.1).

## ELISA

Intracellular cAMP and p-CREB in mHBOs were measured using ELISA kits according to the manufacturer’s instructions (cAMP: MM-61018H2; p-CREB: MM-0006H2; Jiangsu Meimian industrial Co., Ltd.). Absorbance at 450 nm was recorded with a microplate reader (Labsystems Multiskan MS, 352), and concentrations were calculated from standard curves.

## TUNEL assay

Cultures were exposed to 4% PFA at room temperature for 30 min to fix the cells, followed by permeabilization in DPBS containing 0.3% Triton X-100 for 10 min. After two DPBS washes, samples were incubated with 100 µL TUNEL detection solution (Beyotime, C1088) for 30 min at 4 °C in the dark. Subsequently, they were washed twice with DPBS (5 min per wash), and imaging was performed using a confocal laser scanning microscope (LSM800; Carl Zeiss).

## Statistical analysis

Data were analyzed using GraphPad Prism (version 9.5.1). Group differences were evaluated with Student’s t-test for two-group comparisons and one-way ANOVA for multiple groups. Statistical significance was indicated by asterisks: *p < 0.05, **p < 0.01 and ***p < 0.001. Error bars denote standard deviations (SD). Full statistical details are reported in the corresponding figure legends.

## Results

### LRC alleviates liver fibrosis in a CCl_4_-induced mouse model

**FIGURE 1:**
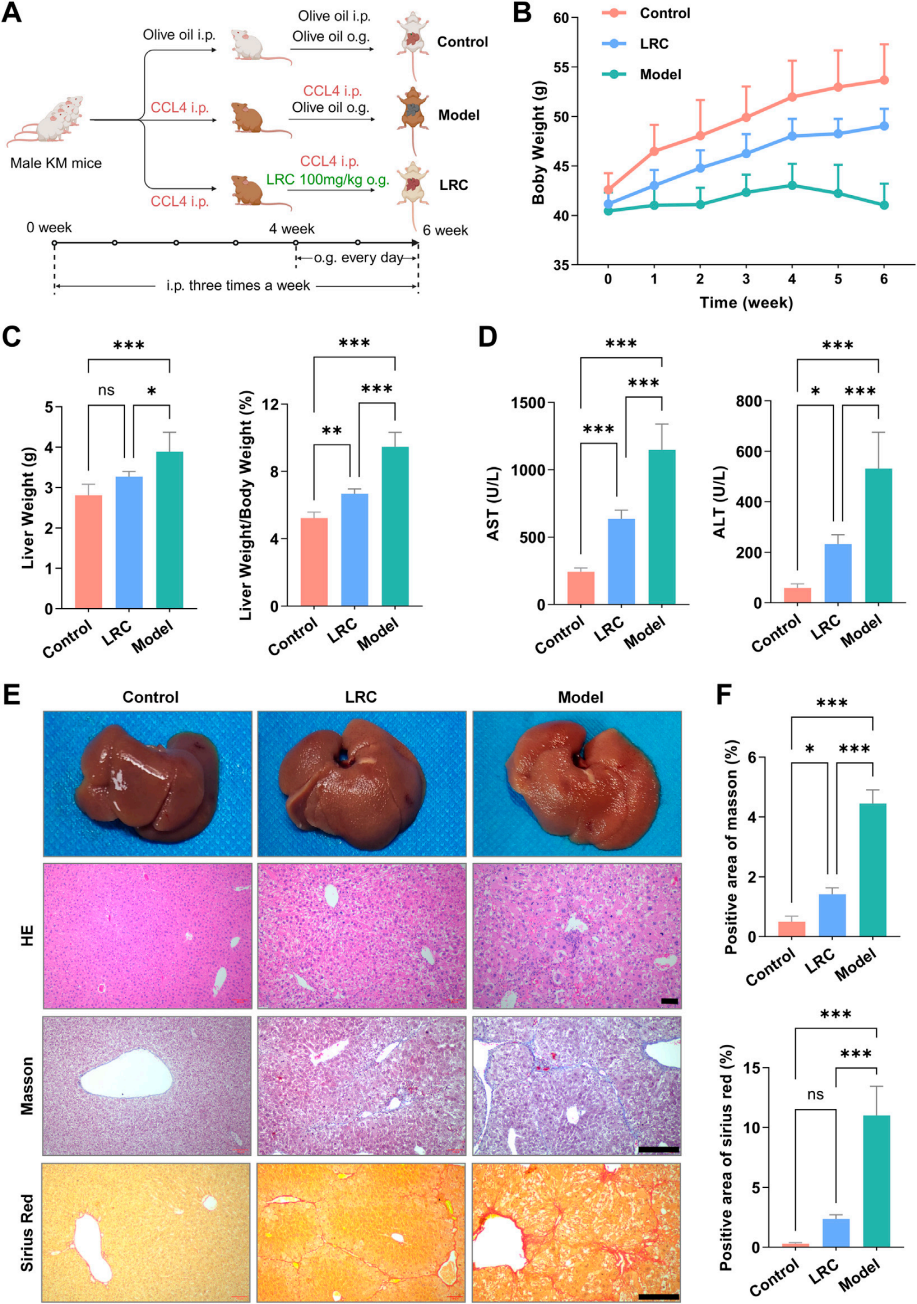
LRC alleviates liver fibrosis in a CCl_4_-induced mouse model. **(A)** Experimental schematic of LRC treatment in mice with CCl4-induced fibrosis. From week 4 to week 6, mice received daily oral gavage of either olive oil (Control) or LRC at 50 mg/kg/day; animals were sacrificed at the end of week 6 (n = 5 per group). Created with BioRender.com. **(B)** Body weight trajectories for the Control, Model, and LRC-treated groups. **(C)** Bar plots at study endpoint: absolute liver weight (left) and liver-to-body weight ratio (right). **(D)** Bar plots of serum levels of AST and ALT at the study endpoint. **(E)** Representative gross liver images and liver histology (H&E, Masson’s trichrome, and Sirius red). **(F)** Bar graphs showing the percentage of positive area for Masson’s trichrome and Sirius red staining across the three groups. Data are presented as the mean ± SD (n = 5 independent experiments). Scale bar = 25 μm. Statistical significance was determined using ANOVA test (ns (not significant), *p < 0.05, **p < 0.01, ***p < 0.001).

**FIGURE 2:**
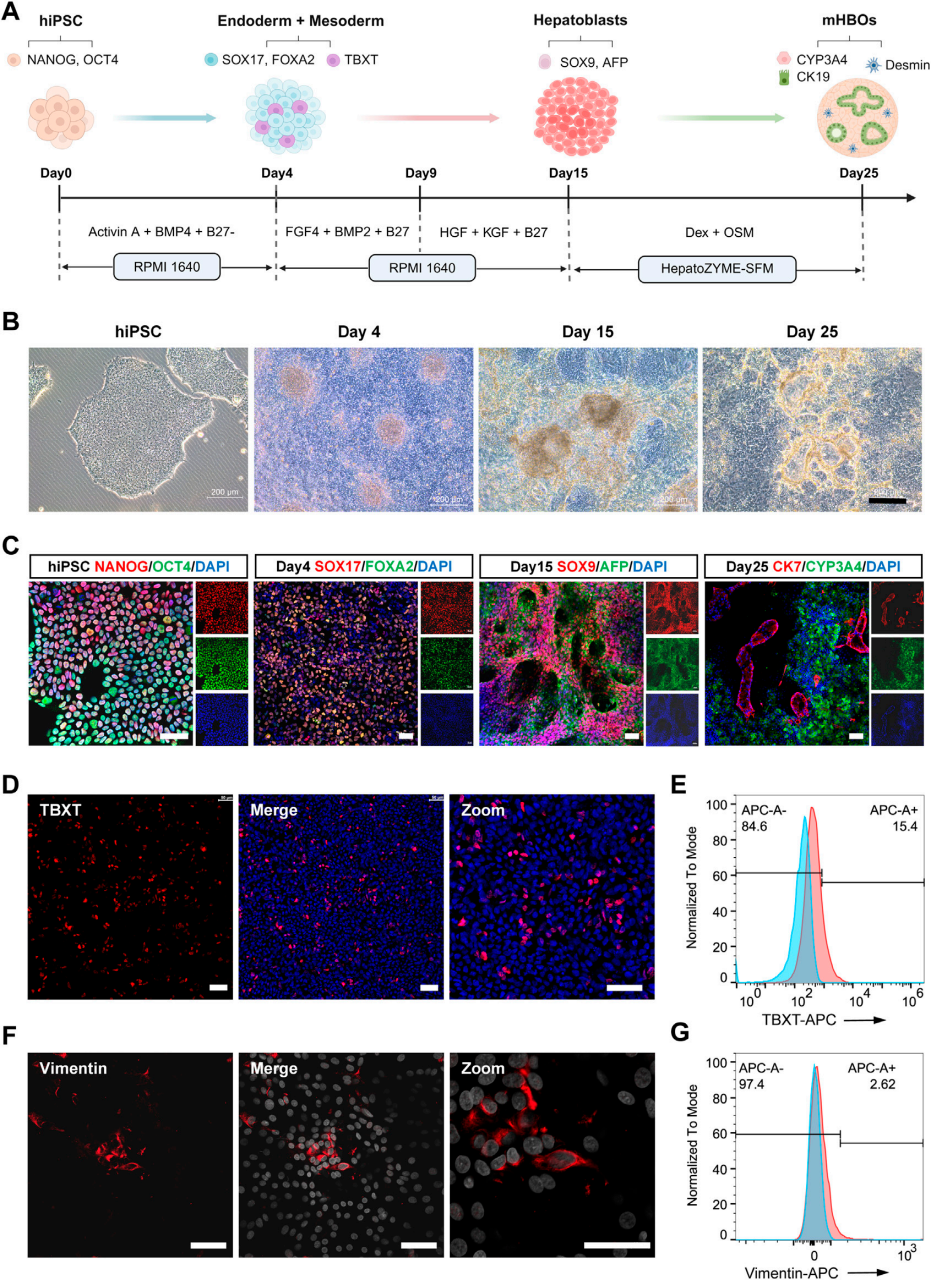
Generation of Multilineage Hepatobiliary Organoids from Human iPSCs. **(A)** Schematic illustrating the differentiation process for mHBOs. Created with BioRender.com. **(B)** Bright-field images show the morphological progression of mHBO differentiation at days 0, 4, 15, and 25 (left to right). Scale bars = 100 µm. **(C)** Immunofluorescence for lineage-specific markers in mHBOs across developmental stages. Day 0: NANOG/OCT4 (iPSCs); Day 4: SOX17/FOXA2 (definitive endoderm); Day 15: SOX9/AFP (hepatoblasts); Day 25: CK7/CYP3A4 (mature cholangiocytes/hepatocytes). **(D)** Immunofluorescence at day 4 demonstrates TBXT-positive mesodermal cells. **(E)** Flow cytometry at day 4 indicates ∼15% TBXT-positive cells. **(F)** Immunofluorescence at day 25 reveals Desmin-positive hepatic stellate cells in mHBOs. **(G)** Flow cytometry identifies 2.6% Desmin-positive cells in mHBOs. All images representative of n ≥ 3 samples.

To assess the therapeutic potential of LRC against liver fibrosis, we established a mouse model by repeated carbon tetrachloride (CCl_4_) administration and treated the animals concomitantly with LRC (Figure 1A; see Methods: Animal Experiments). First, LRC attenuated the CCl_4_-induced reduction in body weight gain, as shown by longitudinal body weight trajectories (Figure 2B), suggesting an improved nutritional status. At the study endpoint, liver mass and serum markers of liver injury were determined. LRC significantly reduced CCl_4_-induced hepatomegaly and decreased serum alanine aminotransferase (ALT) and aspartate aminotransferase (AST) levels (Figures 2C,D).

Histologically, hematoxylin–eosin (H&E) staining showed fibrous septa arising from periportal regions and extending outward to form bridging fibrosis in the model group, with accompanying inflammatory cell infiltration, hepatocyte ballooning, and focal necrosis. In contrast, LRC-treated mice exhibited thinner and less extensive fibrous septa, decreased bridging fibrosis and inflammation, and partial restoration of normal lobular architecture (Figure 2E). In line with these findings, Masson’s trichrome and Sirius Red staining revealed minimal extracellular matrix collagen in controls, abundant deposition in the model group, and a marked reduction following LRC treatment (Figures 2E,F).

### Generation of multilineage hepatobiliary organoids from human iPSCs

Given the species-specific differences in animal models and the inability of monolayer hepatic stellate cell cultures to model the complex pathology of liver fibrosis, we developed more physiologically relevant human multilineage hepatobiliary organoids (mHBOs) to assess the anti-fibrotic efficacy of LRC. Specifically, to generate mHBOs containing mesoderm-derived hepatic stellate cells (HSCs) from a single iPSC line, we followed the protocol described by Wu et al. (2019), retaining a small proportion of mesodermal cells during the definitive endoderm stage. A schematic overview of the differentiation procedure and the associated sequential morphological changes is shown in Figures 2A,B.

Differentiation was validated by stage-specific marker expression using immunofluorescence and flow cytometry (Figures 2C; Supplementary Figure S2A). At the initiation of differentiation, more than 95% of hiPSCs co-expressed the pluripotency factors NANOG and OCT4, ensuring their directed differentiation potential. By day 4, the majority of cells co-expressed the definitive endoderm markers of FOXA2 and SOX17, whereas approximately 15% of cells formed a mesodermal subpopulation expressing TBXT (Figures 2D,E). By day 15, most cells co-expressed SOX9 and AFP, consistent with a bipotent hepatoblast phenotype. By day 25, hepatocytes and cholangiocytes expressed mature lineage-specific markers (CYP3A4 and CK7, respectively), organized into hepatobiliary structures (Figure 2B), produced albumin (Supplementary Figure S2B) and expressed the functional cystic fibrosis transmembrane conductance regulator (CFTR) transporter (Supplementary Figure S2C). Importantly, vimentin-positive HSCs were identified within mature mHBOs (Figures 2F,G), this resident mesenchymal population is essential for modeling hepatic fibrosis.

### LRC alleviates TGF-β–Induced Hepatic Fibrosis in mHBOs

**FIGURE 3:**
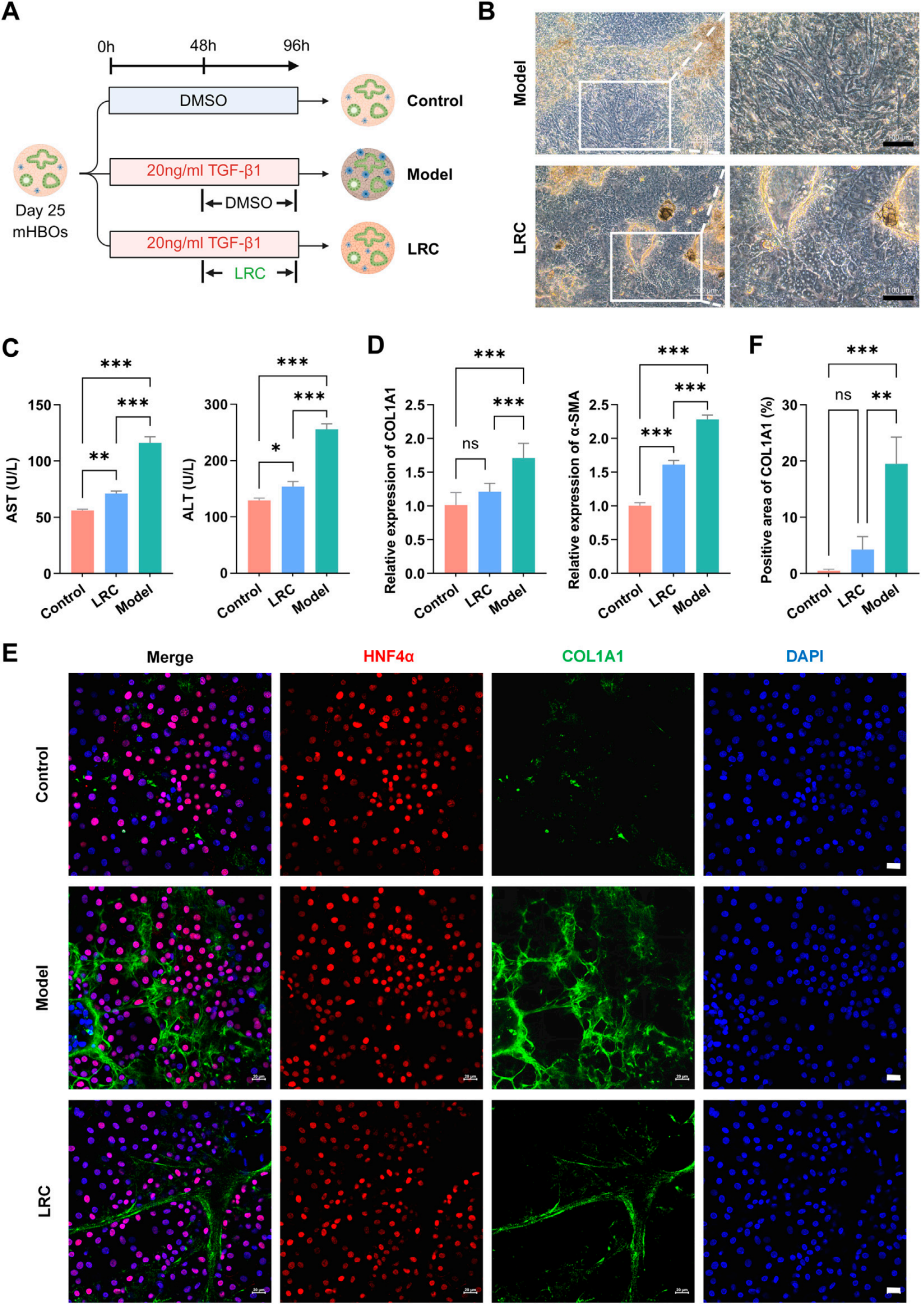
LRC Alleviates TGF-β–Induced Hepatic Fibrosis in mHBOs. **(A)** Schematic of the experimental design for LRC treatment in TGF-β–induced fibrotic mHBOs. The model group received continuous TGF-β (20 ng/mL) stimulation for 96 h; the treatment group was administered 50 μg/mL LRC after 48 h; controls received equivalent DMSO throughout. Created with BioRender.com. **(B)** Bright-field images of mHBOs in the fibrotic model group and the LRC treatment group, scale bars = 50 µm. **(C)** ELISA of AST (left) and ALT (right) in supernatants from the three groups. Data are presented as the mean ± SD (n = 5 independent experiments). **(D)** qPCR for relative expression of *COL1A1* (left) and *α-SMA* (right) in the three groups, normalized to *GAPDH*. Data are presented as the mean ± SD (n = 3 independent experiments). **(E)** Representative immunofluorescence images of COL1A1 deposition in mHBOs from the three groups **(F)** with quantitative analysis of fluorescence area. Data are presented as the mean ± SD (n = 3 independent experiments). Scale bars = 20 µm. Statistical significance was determined using ANOVA test (ns (not significant), *p < 0.05, **p < 0.01, ***p < 0.001). All images representative of n = 3 samples.

We established an organoid fibrosis model by continuously exposing mHBOs to TGF-β, while control organoids were maintained with DMSO. In the treatment group, LRC was administered at the mid-point of TGF-β stimulation. Experimental details are provided in the Methods and depicted in Figure 3A.

Bright-field microscopy showed that 96 h of TGF-β stimulation disrupted organoid architecture, leading to increased granularity and the appearance of refractile deposits in the model group. In contrast, LRC treatment mitigated these morphological alterations and largely preserved normal structural integrity (Figures 2B, 3B). In supernatants, ELISA analysis revealed marked increases in AST and ALT levels in the model group, which were significantly reduced by LRC (Figure 3C). At the transcriptional level, expression of *COL1A1* (a marker of extracellular matrix deposition) and *α-SMA* (a marker of activated HSCs) peaked in the model group (Figure 3D). Quantitative immunofluorescence further confirmed that the model group exhibited the largest fluorescence areas for COL1A1 and α-SMA, while LRC treatment substantially reduced collagen deposition and attenuated α-SMA activation, although not fully to control levels (Figures 3E,F; Supplementary Figure S2A,B).

### Network pharmacological analysis of LRC for hepatic fibrosis

**FIGURE 4:**
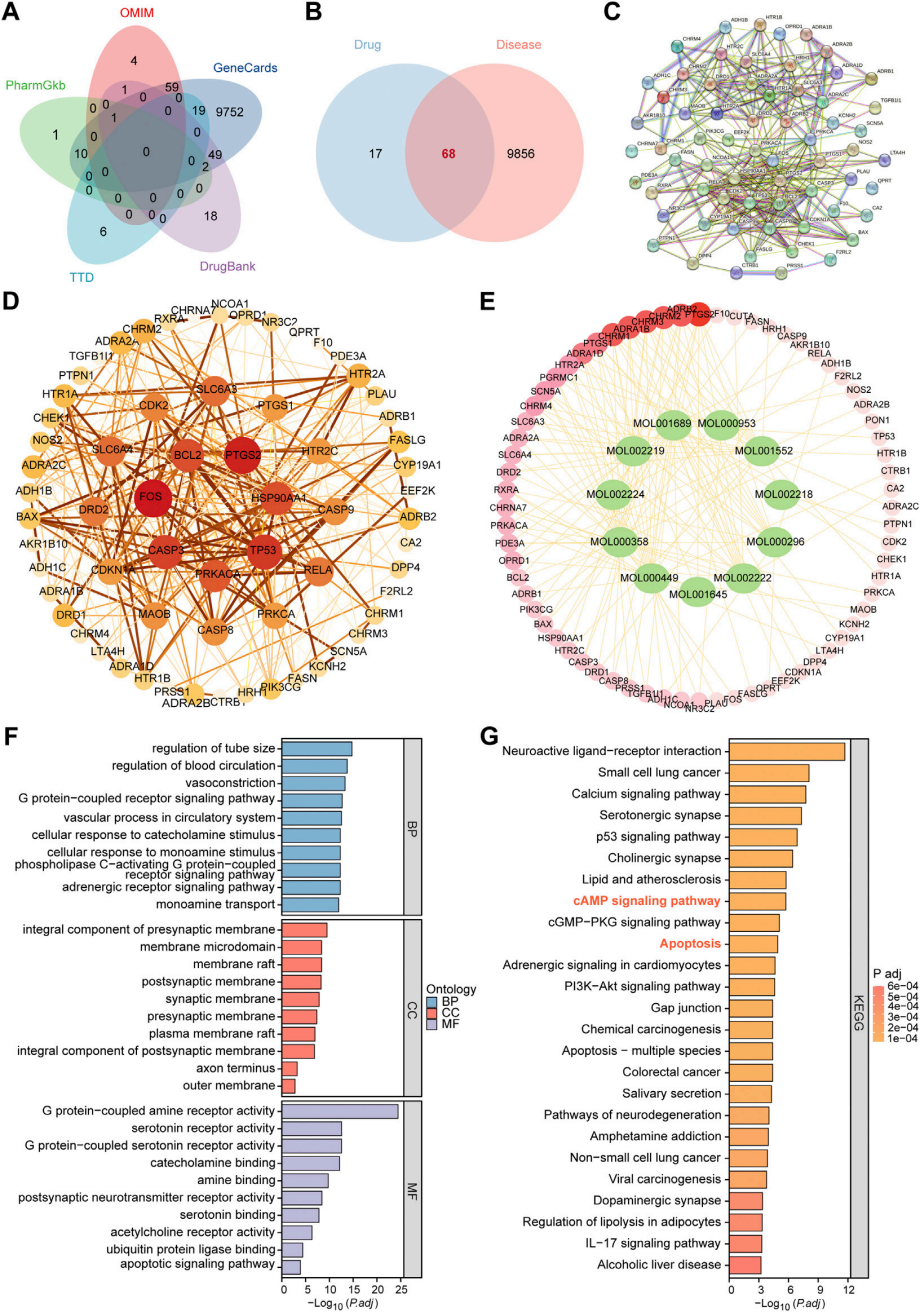
Network Pharmacological Analysis of LRC for Hepatic Fibrosis. **(A)** Venn diagram of the union of hepatic fibrosis–related targets across five databases. **(B)** Venn diagram of the intersection between LRC targets and hepatic fibrosis–related targets. **(C)** PPI network constructed using the STRING database. **(D)** PPI network optimized by node scoring and visualized in Cytoscape. **(E)** Network diagram of LCR active component–target interactions. **(F)** GO enrichment analysis results. **(G)** KEGG pathway enrichment analysis findings.

To identify the active compounds of LRC and their potential antifibrotic targets, we first queried the TCMSP database. Using selection thresholds of oral bioavailability (OB) ≥ 30% and drug-likeness (DL) ≥ 0.18, a total of 13 active compounds (Supplementary Table S1) and 85 associated targets were obtained. In parallel, fibrosis-related targets were collected from GeneCards, OMIM, DrugBank, PharmGKB, and TTD, yielding a combined dataset of 9,924 disease-associated targets (Figure 4A). Comparing the compound-linked targets with the fibrosis target set revealed 68 overlapping genes, identified as potential targets of LRC in hepatic fibrosis (Figure 4B).

Based on the STRING database, we constructed a PPI network for the 68 overlapping targets (Figure 4C). Network centrality was evaluated in Cytoscape using the CytoNCA plugin, and nodes were scored by combined score, with degree value used to indicate relative importance (Figure 4D). Subsequently, a compound-target network integrating the 13 active compounds and these targets was built to identify hub compounds (Figure 4E). Among these, beta-sitosterol (MOL000358) showed the highest connectivity, interacting with 29 key targets, followed by acacetin (MOL001689, 25 targets), stigmasterol (MOL000449, 25 targets), and OIN (MOL001552, 21 targets), suggesting that these constituents may be principal contributors to the anti-fibrotic activity of LRC.

Gene Ontology (GO) enrichment revealed significant biological processes including receptor-driven signaling with adenylate cyclase activation, responses to monoamines/catecholamines, and negative regulation of apoptosis. Enriched cellular components mapped to synaptic membranes and membrane microdomains/rafts that facilitate efficient second-messenger signaling and kinase coupling. Molecular functions emphasized monoamine—particularly serotonin—receptor activities and effector binding, together suggesting receptor-initiated second-messenger pathways linked to apoptosis suppression and antifibrotic effects (Figure 4F). Kyoto Encyclopedia of Genes and Genomes (KEGG) enrichment covered receptor–ligand and calcium-related pathways among the top entries; however, given their relevance to fibrosis, we focused on the cAMP signaling pathway and apoptosis, which together point to a receptor–second-messenger connection with cell-death control (Figure 4G).

Overall, these network pharmacology findings support that LRC may mitigate hepatic fibrosis by modulating apoptosis through a receptor-triggered cAMP-PKA axis.

### LRC activates the cAMP–PKA pathway to inhibit apoptosis

**FIGURE 5:**
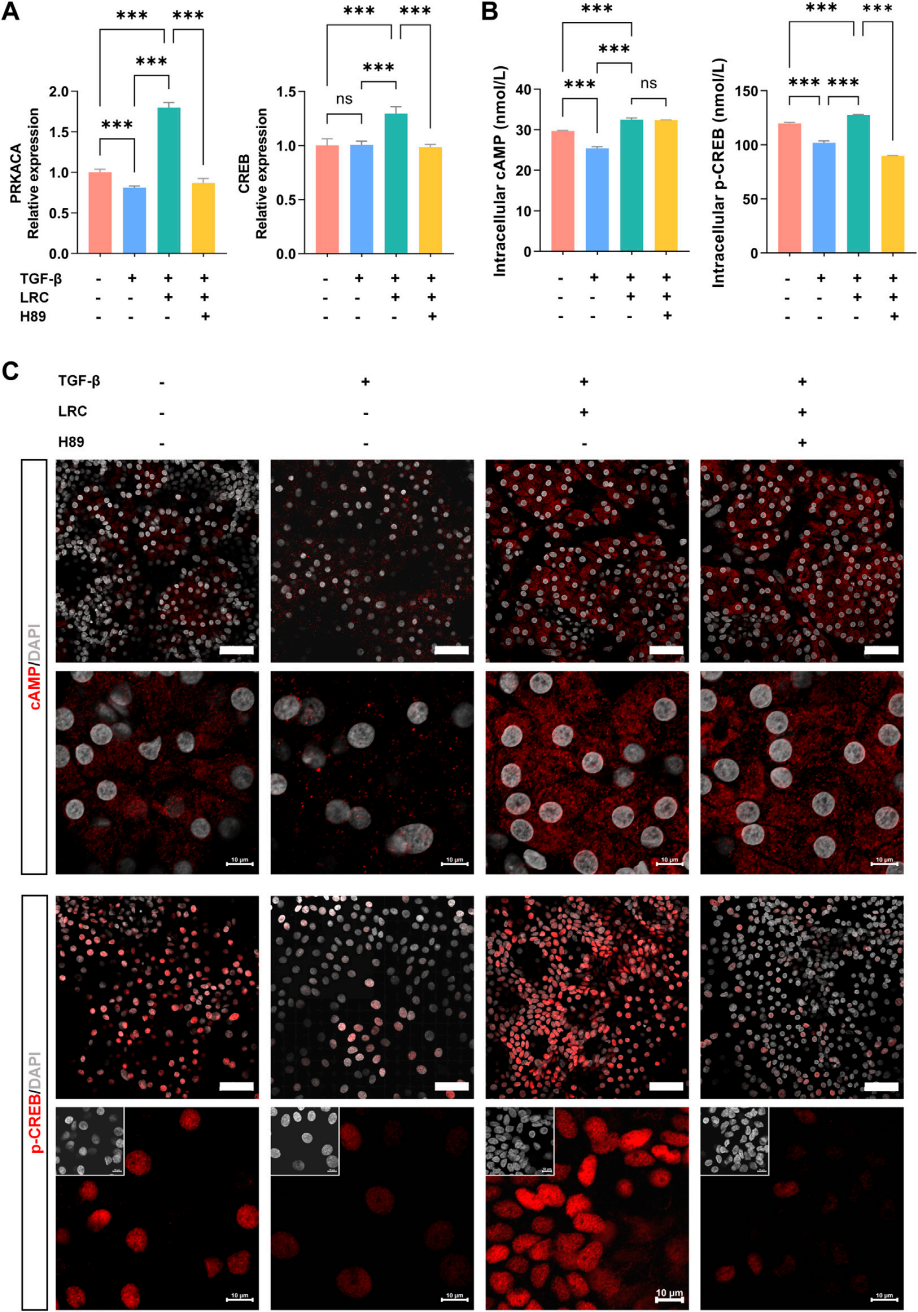
LRC upregulates cAMP–PKA signaling in fibrotic mHBOs. **(A)** qPCR analysis of relative mRNA expression of *PRKACA* and *CREB* across groups, normalized to *GAPDH*. Data are presented as the mean ± SD (n = 3 independent experiments). **(B)** ELISA of intracellular cAMP and p-CREB levels across groups. Data are presented as the mean ± SD (n = 5 independent experiments). **(C)** Representative immunofluorescence images of cAMP and p-CREB in each group, images representative of n = 3 samples. Scale bars = 50 µm. Statistical significance was determined using ANOVA test (ns (not significant), *p < 0.05, **p < 0.01, ***p < 0.001).

To assess whether LRC engages the cAMP–PKA pathway during hepatic fibrosis, mHBOs were pretreated with H89 (a PKA inhibitor) prior to LRC administration. qPCR analysis showed that TGF-β-induced fibrotic models exhibited reduced transcription of *PRKACA* (encoding the catalytic α subunit of PKA), indicating impaired PKA activity. By contrast, *CREB* transcript levels were unchanged; consistent with CREB activation being regulated post-translationally by PKA-mediated phosphorylation. LRC increased the transcript levels of both genes, and these effects were reversed by H89 (Figure 5A).

To further evaluate pathway activity, intracellular cAMP and the downstream effector phosphorylated CREB (p-CREB) were quantified in mHBOs by ELISA. Consistent with impaired PKA signaling, TGF-β reduced cellular cAMP and p-CREB, whereas LRC increased both. As expected for a PRKACA inhibitor, H89 markedly lowered p-CREB with minimal effects on cAMP (Figure 5B). Immunofluorescence analysis yielded concordant findings: quantification of mean fluorescence per cell showed the lowest cAMP and p-CREB signals in TGF-β–treated mHBOs. In LRC-treated cultures, cAMP intensity remained similar with or without H89, but p-CREB was substantially higher without the inhibitor. Representative images further show that LRC increases nuclear p-CREB enrichment, with higher intranuclear fluorescence at the single-cell level; this enrichment is reduced by H89 (Figures 5C; Supplementary Figure S3A, S42B). These findings support activation of the cAMP–PKA axis by LRC in fibrotic liver organoids.

**FIGURE 6:**
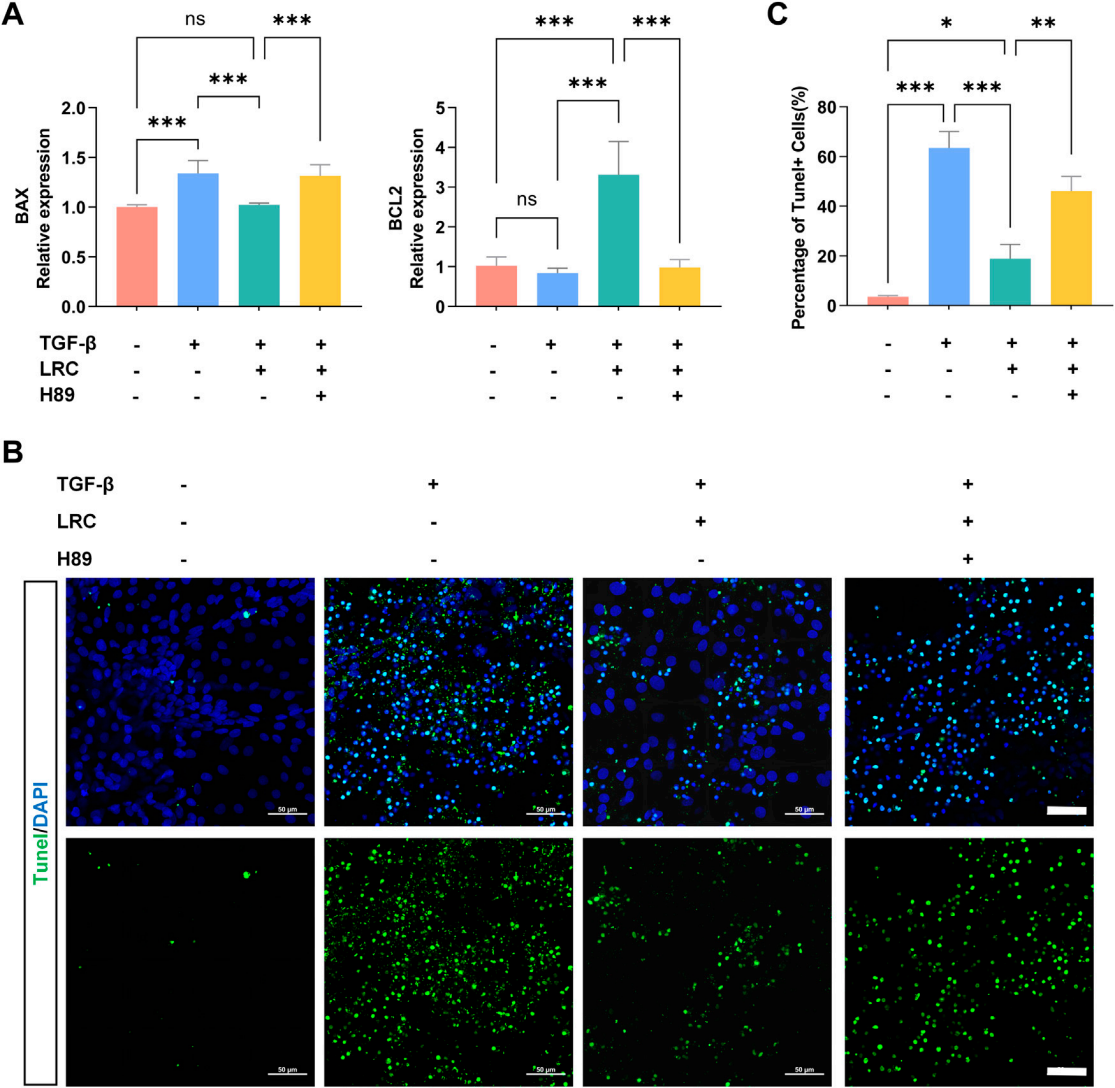
LRC inhibits apoptosis via upregulation of the cAMP–PKA pathway. **(A)** qPCR analysis of relative *BAX* and *BCL2* expression across groups. Data are presented as the mean ± SD (n = 3 independent experiments). **(B)** Representative TUNEL staining images for each group and **(C)** quantification of TUNEL-positive cells (%), images representative of n = 3 samples. Data are presented as the mean ± SD (n = 3 independent experiments). Scale bars = 50 µm. Statistical significance was determined using ANOVA test (ns (not significant), *p < 0.05, **p < 0.01, ***p < 0.001).

Next, we examined whether the cAMP–PKA pathway is linked to apoptosis in mHBOs. qPCR analysis of the pro-apoptotic gene *BAX* and the anti-apoptotic gene *BCL2* showed that TGF-β increased *BAX* expression, whereas LRC markedly decreased *BAX* and elevated *BCL2*; these effects were reversed by H89 pretreatment (Figure 6A). TUNEL staining revealed abundant fluorescently positive cells in TGF-β–induced mHBOs, indicating widespread apoptosis, consistent with characteristic morphological changes observed under bright-field microscopy. LRC reduced the number of TUNEL-positive cells, and this anti-apoptotic effect was suppressed by H89 (Figures 6B,C). Furthermore, under H89 pretreatment, the therapeutic benefits of LRC in reducing collagen fiber deposition and inhibiting HSC activation were attenuated (Supplementary Figure S3B,C). We also replicated the key validation experiments in mHBOs derived from an independent iPSC line (Q-iPS-10) and obtained concordant results, thereby mitigating potential batch effects (Supplementary Figure S5).

Collectively, these results demonstrate that LRC attenuates TGF-β–induced fibrosis in mHBOs by suppressing parenchymal apoptosis via activation of the cAMP–PKA signaling pathway.

## Discussion

Liver fibrosis represents a major clinical challenge with no approved anti-fibrotic drugs currently available, making discovery of effective agents an urgent need (Parola et al., 2024; Juanola et al., 2025; Mohammed et al., 2023). A notable strength of this study is the use of a human mHBOs model incorporating mesoderm-derived HSCs generated via synchronous co-differentiation from a single hiPSC source (Figure 2). This design preserves developmental lineage-lineage interactions and yields organoids capable of exhibiting robust and sustained fibrotic responses upon TGF-β stimulation (Figures 2A,B). Importantly, most reported multilineage hepatic organoid systems typically rely on separately differentiating each cell lineage to maturity before mixing—a strategy that often results in marked heterogeneity in cell maturation and functional status (Kim et al., 2023; Akbari et al., 2019; Moss et al., 2025; Chi et al., 2025; Thompson and Takebe, 2020). Additionally, such assembled models suffer from considerable batch-to-batch variability and limited ability to consistently induce and maintain fibrotic phenotypes, thereby constraining their utility in mechanistic studies and anti-fibrotic drug screening. In the present study, we combined *in vivo* and *in vitro* approaches to provide comprehensive evidence that *Lycii Radicis* Cortex (LRC) exerts potent anti-fibrotic effects. Our findings expand the pharmacological profile of LRC in hepatic fibrosis, bridging a translational gap between conventional rodent models and human-relevant systems.

In a CCl_4_-induced murine model, LRC administration ameliorated liver injury indicated by improved body weight gain, reduced serum ALT and AST, and attenuation of histological markers of fibrosis including collagen deposition (Figures 2B–F). Extending this observation to a human multilineage hepatobiliary organoids (mHBOs) model incorporating hepatic stellate cells (HSCs), we demonstrated that LRC significantly mitigated TGF-β–induced ECM accumulation and HSC activation, paralleled by decreased organoid structural disruption and improved biochemical injury markers (Figures 3B–F; Supplementary Figure S2A,B). To strengthen methodological rigor and translational relevance, we replicated all key mHBO experiments in an independent hiPSC line under identical protocols; results were consistent across lines, reducing the influence of cell line–specific and batch-specific effects and supporting LRC’s antifibrotic activity (Supplementary Figure S5).

Mechanistically, network pharmacology implicated 13 bioactive compounds, with beta-sitosterol, acacetin, and stigmasterol emerging as potential lead constituents via high target connectivity (Figures 4A–E). GO and KEGG enrichment analyses converged on pathways related to receptor-mediated cAMP signaling and apoptosis regulation (Figures 4F,G), indicating that modulation of cyclic nucleotide signaling cascades could be a central axis through which LRC mediates its anti-fibrotic effects. Notably, cAMP is recognized as a second messenger capable of integrating upstream receptor activation with downstream transcriptional programs, including those governing cell survival and matrix turnover (Musheshe et al., 2018). Beta-sitosterol and stigmasterol have been reported to mitigate hepatic fibrosis via modulation of MAPK, NF-κB, and NLRP3 pathways (Devaraj et al., 2020; Wang X. et al., 2025; Han et al., 2024) and intersect with cAMP-related signaling in other systems (beta--sitosterol: cAMP/NF-κB in osteoclasts; stigmasterol: cAMP-CREB in the nervous system) (Guo et al., 2025; Haque et al., 2018), supporting a mechanistic link to the cAMP–PKA–CREB activation observed here. Acacetin, a hepatoprotective flavone, may further augment this axis (Zhou et al., 2021; Chen and Gao, 2024), as flavone scaffolds can elevate intracellular cAMP via phosphodiesterase inhibition (Cormier et al., 2018; Hamsalakshmi et al., 2022). Although constituent levels and exposure were not quantified, phytochemical studies identify sterols and flavonoids as measurable LRC components (Yang et al., 2017). Together, these observations provide a focused molecular rationale for LRC’s antifibrotic activity.

Mechanistic validation in the mHBO model confirmed that LRC activates the cAMP–PKA pathway, as evidenced by increased *PRKACA* transcription, elevated intracellular cAMP levels, and enhanced phosphorylation of CREB (Figure 5; Supplementary Figure S3A). Importantly, pharmacological inhibition of PKA with H89 abolished these effects and reversed LRC-mediated protection against fibrosis and parenchymal apoptosis (Figure 6; Supplementary Figure S3B,C). This is consistent with literature demonstrating that cAMP–PKA signaling can suppress HSC activation, promote matrix degradation, and inhibit pro-fibrotic gene expression (Wójcik-Pszczoła et al., 2020; Weng et al., 2015), partly via phosphorylated CREB’s transcriptional regulation of apoptosis-related genes such as *BCL2* and *BAX* (Wahlang et al., 2018; Li et al., 2025; Wang Q. et al., 2025; Zhang et al., 2024; Yang et al., 2015). These effects are thought to occur in part through phosphorylated CREB’s transcriptional regulation of apoptosis-related genes such as *BCL2* and *BAX*, thereby re-balancing cell fate toward resolution of fibrotic injury. Complementing this, GO/KEGG enrichment included G protein-coupled receptor (GPCR) signaling and adenylyl cyclase (AC)–related terms, and the PPI network featured ADRB1/ADRB2/CHRM1, suggesting potential receptor-level input into the cAMP-PKA-CREB axis (Ripoll et al., 2025). While our data demonstrate PKA dependence (H89-sensitive), GPCR/AC involvement was not directly tested.

This study has several limitations. We used only male mice, precluding assessment of sex-dependent variability and limiting generalizability across sexes. The *in vivo* design lacked a non-fibrotic LRC-only group, limiting assessment of baseline effects of LRC on hepatic morphology, metabolic parameters, and signaling (e.g., cAMP–PKA) in the absence of injury. Finally, although the data implicate the cAMP–PKA axis, contributions from upstream or parallel pathways (e.g., GPCR–adenylyl cyclase) cannot be excluded. Future work will include age-matched female cohorts, add an LRC-only group, and test receptor-mediated inputs with targeted pharmacologic and genetic perturbations.

In conclusion, this study demonstrates that LRC attenuates hepatic fibrosis by suppressing parenchymal apoptosis via activation of the cAMP–PKA signaling pathway, substantiated through both murine and human organoid models. Beyond revealing a novel anti-fibrotic mechanism for a traditional medicinal material, our work highlights the utility of mHBOs as an effective translational platform for natural product research, mechanistic studies, and drug discovery in chronic liver disease.

## Data Availability

The original contributions presented in the study are included in the article/Supplementary Material, further inquiries can be directed to the corresponding authors.
