# Supplementary material for: Lycii radicis cortex alleviates fibrosis in hiPSC-derived multilineage hepatic organoids via the cAMP-PKA pathway
Source: Front Pharmacol. 2025 Nov 25;16:1730255. doi: 10.3389/fphar.2025.1730255 (PMC12687450; doi:10.3389/fphar.2025.1730255)
Supplement: Supplementary file 1 [file Supplementaryfile2.docx]

**Supplementary Table 1. Active compounds of LRC**

| **Molecule ID** | **Molecule Name** | **OB ≥ 30%** | **DL ≥ 0.18** |
| --- | --- | --- | --- |
| MOL001552 | OIN | 45.97 | 0.19 |
| MOL001645 | Linoleyl acetate | 42.1 | 0.2 |
| MOL001689 | acacetin | 34.97 | 0.24 |
| MOL001790 | Linarin | 39.84 | 0.71 |
| MOL002218 | scopolin | 56.45 | 0.39 |
| MOL002219 | Atropine | 34.53 | 0.21 |
| MOL002222 | sugiol | 36.11 | 0.28 |
| MOL002224 | aurantiamide acetate | 58.38 | 0.59 |
| MOL002228 | Kulactone | 45.44 | 0.82 |
| MOL000296 | hederagenin | 36.91 | 0.75 |
| MOL000358 | beta-sitosterol | 36.91 | 0.75 |
| MOL000449 | Stigmasterol | 43.83 | 0.76 |
| MOL000953 | CLR | 37.87 | 0.68 |

**Supplementary Table 2. Quantitative PCR primer list**

| **Gene** | **Forward** | **Reverse** |
| --- | --- | --- |
| *GAPDH* | GGAGCGAGATCCCTCCAAAAT | GGCTGTTGTCATACTTCTCATGG |
| *COL1A1* | GAGGGCCAAGACGAAGACATC | CAGATCACGTCATCGCACAAC |
| *α-SMA* | CTATGAGGGCTATGCCTTGCC | GCTCAGCAGTAGTAACGAAGGA |
| *BAX* | TCAGGATGCGTCCACCAAGAAG | TGTGTCCACGGCGGCAATCATC |
| *BCL2* | ATCGCCCTGTGGATGACTGAGT | GCCAGGAGAAATCAAACAGAGGC |
| *PRKACA* | AGCCCACTTGGATCAGTTTGA | GTTCCCGGTCTCCTTGTGT |
| *CREB* | GACCACTGATGGACAGCAGATC | GAGGATGCCATAACAACTCCAGG |

**Supplementary Table 3. Antibodies used in immunofluorescence analyses**

| **Target** | **Supplier** | **Host** | **Cat No.** |
| --- | --- | --- | --- |
| NANOG | Abcam | Rabbit | Ab109250 |
| OCT4 | Abcam | Mouse | Ab184665 |
| SOX17 | Gene Tex | Mouse | GTX83580 |
| FOXA2 | Abcam | Rabbit | Ab214449 |
| TBXT | Abcam | Rabbit | Ab209665 |
| HNF4α | CST | Rabbit | 3113s |
| HNF4α | RD | Mouse | MAB4605 |
| AFP | RD | Mouse | MAB1368 |
| SOX9 | RD | Goat | AF3075 |
| CK7 | abcam | Rabbit | ab68459 |
| CYP3A4 | PGT | Mouse | 67110-1-Ig |
| ALB | abcam | Rabbit | ab207327 |
| CFTR | CST | Rabbit | 78335s |
| α-SMA | CST | Rabbit | 19245S |
| COL1A1 | CST | Mouse | 66948S |
| cAMP | PGT | Rabbit | 12009-1-AP |
| p-CREB | CST | Rabbit | 9198T |
| Donkey anti-Rabbit IgG  Alexa Fluor 488 | Invitrogen | Donkey | A-21206 |
| Goat anti-Rabbit IgG  Alexa Fluor Plus 594 | Invitrogen | Donkey | A-32740 |
| Donkey anti-Mouse IgG  Alexa Fluor 488 | Invitrogen | Donkey | A-21202 |
| Donkey anti-Mouse IgG  Alexa Fluor 594 | Invitrogen | Donkey | A-32744 |
| Donkey anti-Goat IgG  Alexa Fluor 488 | Invitrogen | Donkey | A-11055 |

| Donkey anti-Goat IgG  Alexa Fluor 594 | Invitrogen | Donkey | A-11058 |
| --- | --- | --- | --- |
